# Supplementary material for: Perceptions of homogeneity reproduction in health sciences academia
Source: PLoS One. 2026 Oct 28;21(10):e0345194. doi: 10.1371/journal.pone.0345194 (PMC13716225; doi:10.1371/journal.pone.0345194)
Supplement: Supplementary file 1 [file pone.0345194.s001.docx]

# S1 Appendix. Interview guide

Interview schedule for doctoral students, postdocs & professors on the perception of opportunities and challenges for the academic career of marginalised groups

1. Narrative-generating opening question:

- Perhaps you could start by telling me how it was that you ended up in academia and what you found attractive about academia and what disillusioned you over time? (feel free to go as far as you like and tell me freely)

2. Working conditions:

- What kind of work do you do? (e.g. laboratory work, clinical work, teaching)
- How would you describe your working conditions?
- When do you do your work?
- Do you do other (voluntary) university work in addition to your paid work? (e.g. committee work, project coordination, involvement in other projects, teaching, academic self-administration)

3. University support:

- How would you rate university support (support from supervisors, professors, superiors, institute) during your time in academia?
- How do you explain the current level of university support?
- Where would you like more support?
- What impact does the current level of university support have on you in academia?
- Looking back on your time in academia so far, how would you rate the level of support you have received from the university?

4. Funding:

- If applicable, how do you fund your academic career? (e.g. paid work alongside a doctorate)
- To what extent does the financing of your academic career represent a stress factor for you?
- To what extent does the funding situation influence your academic work? (e.g. your productivity of your work?)
- Looking back on your time in academia so far, how would you rate your financial situation?
- To what extent do financial considerations play a role in your future plans to work in academia?

5. Self-confidence and self-efficacy

- How confident and self-assured do you feel about your work in science?
- What are factors that make you feel confident/insecure? (Question about precariousness in science, funding, discrimination)
- To what extent does your self-confidence/insecurity affect your academic career (e.g. productivity)?
- Which personal qualities (e.g. resilience, hope, optimism) have helped you in particular with your scientific work?

6 External responsibilities

- To what extent do your family/friends support you in your work in science?
- If applicable, how do you explain the lack of support?
- Do you have other responsibilities in your family or circle of friends in addition to your academic work? (e.g. care tasks)
- To what extent do these responsibilities influence your academic work?
- When you look back on your time in academia so far, how would you rate responsibilities in your family or circle of friends?

7. Health

- What is your health situation (physical and mental)?
- To what extent do the contextual conditions of your academic career (e.g. lack of supervision, fear of the future) lead to stress, anxiety and other physical and mental stressors?

8. Future professional life:

- What are your professional expectations for the future?
- What fears and positive expectations are associated with these ideas?
- To what extent do factors such as gender, socio-economic background, external responsibilities (e.g. care tasks) etc. influence your professional expectations/enjoyments/expectations? influence your professional expectations/joys/fears for the future?
- To what extent do structural factors in science (e.g. precariousness, working hours) influence your future career expectations?

9. Discrimination and exclusion

- To what extent does your gender, sexual orientation, race, socio-economic background (and other identities of yours) influence your scientific life (e.g. productivity)?
- Would you say that you have ever been discriminated against during your time in academia and if so, what kind of discrimination was it?
- Do you know other people who have had similar experiences?
- How could this problem be tackled?
- What would have to change in academia/what structures would have to be created so that such forms of discrimination no longer happen?
