# Supplementary material for: Perceptions of homogeneity reproduction in health sciences academia
Source: PLoS One. 2026 Oct 28;21(10):e0345194. doi: 10.1371/journal.pone.0345194 (PMC13716225; doi:10.1371/journal.pone.0345194)
Supplement: Supplementary file 2 [file pone.0345194.s002.docx]

# S2 Appendix. Coding tree example

personal background

getting into academia

academic family

connections to professors

genuine academic/research interest

future plans

fulfilling requirements for next career steps

work conditions

(precarious) work conditions

responsibility for colleagues & students

responsibility/carework for colleagues/compassionate leadership

teaching & supervision

PhD/Dr. med

insecurity

self-reliance

AdvanceAcademia

flexible work

financial precarity

supervision

luck with supervisor

female role models

extra semester

capitalisation of academia

unreliability

Wissenschaftszeitvertragsgesetz

multiple expectations

performance pressure

time pressure

precarious contract

uncertain funding

uncertainty of getting funding whilst having family

political & voluntary work

combining clinical work, research & teaching

research-based clinical work

coping with family responsibilities

lacking experience in parts of clinical/academic work due to constraint time inputs

clinician scientist

enjoying diversity in tasks

being torn between academia/clinical work

flexibility through academic work

coping mechanisms to work conditions

resilience

resilience through psychological/psychiatric education

learning from colleagues

good networking

setting boundaries

benefits of working in academia

stable contracts/good conditions - prof.

flat hierarchies

diversity in tasks

flexible working hours

diversity & discrimination

Nature of discrimination

gender

parental leave

career detriments

neglecting other diversity domains for gender

preferring men over women

female only team

appearance as”projection surface”

white (old) cis-male in leading positions

harassment from men

career advantages through female quota

caring for children

relying on wife for carework

lacking basic conditions for children

lacking 'rationality' in career

ambivalence between time for family and career

ambivalence between wanting to care for children and not reducing at work

being less flexible in chosing work locations

working hours

fighting about who reduces working hours

reducing working hours for family

prioritising clinical over academic work

slowing down career

support from family and babysitters

support as resilience

working with women

ideas to improve the system

establishing positions for (more) diversity officers

offering childcare

hierarchy between professions

carework

elderly

children

class

microaggression

queerness (sex)

enjoying other privileges (e.g. whiteness; coolness)

being transparent to be less controversial

performance/going beyond limits as compensation for queerness

queerness (sexuality)

(re)considering openness about queerness

exclusive heteronormative networks

neurodiversity/psychol. factors

mental consequences of exclusion

(fear of) being weird/nonconforming

imposter syndrome

trauma

burnout

ADHD

fear

disabilities

increased barriers to work/physical barriers

ethnicity

white degree courses

denying competence

racism within teaching

white networks

Consequences and coping mechanisms

exit

compensation through performance

attempting conformity
