# Supplementary material for: Perceptions of homogeneity reproduction in health sciences academia
Source: PLoS One. 2026 Oct 28;21(10):e0345194. doi: 10.1371/journal.pone.0345194 (PMC13716225; doi:10.1371/journal.pone.0345194)
Supplement: Supplementary file 3 [file pone.0345194.s003.docx]

# S1 Table. Recruitment matrix

| **Dimension** | **Strata** | **Target** |
| --- | --- | --- |
| Institutional role | Doctoral students  PhD supervisors | 4 to 6  4 to 6 |
| Gender | Women  Men  Non-binary  Another gender identity | >50% overall and within each role  >30% overall  At least 1  At least 1 |
| Career stage | Early (postdoc/junior group lead)  Mid  Senior  Prof | At least 1  At least 1  At least 1  At least 1 |
| Student stage | Early  Mid  Late | At least 1  At least 1  At least 1 |
| Clinical/disciplinary context | Clinical departments  Biomedical  Public health | At least 1  At least 1  At least 1 |
| Contract | Fixed-term  Permanent  Stipend  External funding | At least 1  At least 1  At least 1  At least 1 |
| Care responsibilities | Child  Elderly  Other | At least 1  At least 1  At least 1 |
| Migration background | At least 1  At least 1 | At least 1  At least 2 |
| Disability/Chronic Illness | Disability  Longterm condition | At least 1  At least 1 |
| Neurodiversity | Self-identified neurodivergent | At least 1 |
| Sexual orientation | LGBTQIA+ | At least 1 |
| International mobility | >6 months mobility or strong expectation | At least 1 |
