# Supplementary material for: Perceptions of homogeneity reproduction in health sciences academia
Source: PLoS One. 2026 Oct 28;21(10):e0345194. doi: 10.1371/journal.pone.0345194 (PMC13716225; doi:10.1371/journal.pone.0345194)
Supplement: Supplementary file 5 [file pone.0345194.s006.docx]

**Response to Editor and Reviewers**

PLOS ONE manuscript PONE-D-26-10324
Perceptions of homogeneity reproduction in health sciences academia

Dear Dr Zhou,

We thank you and the reviewers for the careful and constructive assessment of our manuscript. We have revised the paper throughout. The principal changes are summarised below, followed by a point-by-point response. Section names are used instead of page and line numbers because pagination may change during production.

**Summary of revisions**

- Renumbered all 62 references in order of first appearance and reordered the reference list.
- Clarified the analytical novelty of “anticipatory compliance” relative to covering, passing, impression management, minority stress, and self-surveillance.
- Separated participant accounts, theoretical interpretation, and contextual evidence more explicitly throughout the Results and Discussion.
- Tempered the self-reinforcing-cycle claim and other conclusions to reflect the exploratory, single-institution design.
- Expanded the Data Analysis section with a quotation-to-code-to-theme audit-trail example and strengthened the limitations of snowball recruitment.
- Revised the Data Availability Statement to identify the responsible institution, contact address, materials potentially available, and governance conditions; removed funding text from the manuscript; and blinded the verified grant number and recipient details across all submission documents, reserving them for the online Funding Information and Financial Disclosure fields.

**Journal requirements**

**1. PLOS ONE style and file namin**g

**Comment:** *Please ensure that the manuscript meets PLOS ONE style requirements, including file naming.*

**Response:** We reviewed the revised manuscript against the current PLOS ONE submission guidance. The title page, manuscript organisation, supporting-information captions, and Vancouver-style numbered citations have been checked. The submission files are being prepared with the manuscript ID and clear role labels for the clean manuscript, tracked-changes manuscript, and response letter.

**2. Funding Information and Financial Disclosure**

**Comment:** *The grant information in the Funding Information and Financial Disclosure sections does not match; provide the correct grant number(s).*

**Response:** The verified award details will be entered identically in the Funding Information and Financial Disclosure fields at resubmission. The grant number and recipient details have been deliberately blinded across all submission documents—including the clean manuscript, tracked-changes manuscript, supporting files, and this response letter—and will appear only in the online submission fields.

**3. Competing interests**

**Comment:** *Replace “None known” with the journal’s required declaration and include it in the cover letter.*

**Response:** We confirm that the authors have no competing interests. The online form and cover letter will state: “The authors have declared that no competing interests exist.”

**4. Remove funding information from the manuscript**

**Comment:** *Funding information should not appear anywhere in the manuscript.*

**Response: Confirmed. The verified grant number and recipient details have been deliberately blinded across all submission documents. Neither the clean manuscript nor the tracked-changes manuscript contains a funding or sponsorship statement. The verified award details will appear only in the online Funding Information and Financial Disclosure fields.**

**5. Data Availability Statement**

**Comment:** *Identify the third-party contact or institution and provide contact details.*

**Response:** The Data Availability Statement now identifies the Ethics Committee of Charité – Universitätsmedizin Berlin, gives the contact address ethikkommission@charite.de, explains that full transcripts cannot be released, identifies the de-identified excerpts and coding tree that are available, and describes the review, consent-compatibility, ethics, and data-use-agreement conditions for requests for additional anonymised excerpts.

**6. Ethics statement**

**Comment:** *The ethics statement should appear only in the Methods section.*

**Response:** Confirmed. The ethics approval and consent statement appears only under Ethical considerations in Materials and methods. It is not repeated elsewhere.

**7. Suggested citations**

**Comment:** *Review any suggested publications for relevance; citation is not mandatory unless the editor requires it.*

**Response:** Noted. Neither reviewer requested a specific additional citation. No citation was added solely in response to reviewer pressure.

**Reviewer** 1

**1. Reference numbering**

**Comment:** *Reference numbering is inconsistent; for example, citation [50] appears after [7] in the Introduction.*

**Response:** Corrected. We recalculated first appearance across the fully revised manuscript, renumbered every in-text citation, and reordered the reference list. The first appearances now run sequentially from [1] to [62], with no uncited or out-of-range references.

**2. Influence of snowball sampling**

**Comment:** *Explain how snowball sampling may have influenced the findings, particularly by reaching people who are more connected within academic networks.*

**Response:** We expanded the Limitations section. It now explains that professional referrals may have disproportionately reached people who remained connected enough to academic networks to be approached, while people who had disengaged, lacked such ties, or experienced the most acute exclusion may be underrepresented. We also explain that the themes may therefore emphasise negotiating inclusion from within academia rather than complete exclusion or exit.

**3. Overgeneralised conclusion**

**Comment:** *The statement that exclusion is the mechanism through which homogeneity is reproduced is broader than the data support, particularly without racially or ethnically minoritised participants.*

**Response:** We revised the opening of the Conclusions to specify the single-institution sample and to present the relationship as participants’ perceptions: “participants’ accounts suggest that external exclusion and self-directed responses are interconnected processes through which they perceived social homogeneity to be reproduced.” The Limitations section also now states explicitly that the study cannot substantively characterise racialised, ethnic, or physical-disability dimensions of exclusion.

**4. Self-reinforcing cycle**

**Comment:** *Present the self-reinforcing cycle more cautiously as a possible or exploratory interpretation.*

**Response:** We revised the Abstract and Conclusions. The cycle is now described as “an exploratory interpretation of a possible self-reinforcing cycle,” and the Conclusions explicitly state that it is an interpretive model to be tested, not a causal effect established by this study.

**5. Analytic audit trail**

**Comment:** *Add a brief example showing how a quotation was coded and developed into a theme.*

**Response:** We added a concrete example to Data analysis. Interview 8’s phrase “reduce the projection surface” was initially coded as “appearance as ‘projection surface’”; comparison with “(fear of) being weird/nonconforming” and “attempting conformity,” together with memoing and analytic dialogue, led to its grouping with identity-management and self-exclusion practices and its contribution to the higher-order theme of anticipatory compliance. S3 Appendix remains the extended coding-tree example.

**Reviewer** 2

**1. Novelty and construct differentiation**

**Comment:** *Clarify what is analytically novel about anticipatory compliance and how it differs from covering, passing, self-surveillance, impression management, and minority stress.*

**Response:** We added an explicit construct-differentiation paragraph at the start of Results and Discussion. Covering, passing, and impression management are distinguished by what is concealed or presented; minority stress by the broader stress process; and self-surveillance by internalised monitoring. Anticipatory compliance is defined instead as the temporal and relational logic linking partial, cross-domain practices: pre-emptive adjustment of identity, behaviour, effort, or career plans before a specific act of exclusion occurs, while retaining the external conditions that make those adaptations intelligible.

**2. Participant perception, interpretation, and structural inference**

**Comment:** *Separate participant narratives, theoretical interpretation, and external statistical evidence more clearly to avoid overstating structural conclusions.*

**Response:** We added a framing paragraph stating that participant accounts are the empirical basis of the analysis and distinguishing three levels of claim: participant perception, theoretical interpretation, and published evidence used only to situate the institutional context. We also revised the passages on gender, class, ableism, and neurodiversity to label external studies as contextual rather than corroborative, and to state that they do not establish prevalence or causality in this sample.

**3. Sample boundaries and interpretive caution**

**Comment:** *Maintain caution given the small, snowball-recruited, faculty-heavy sample and the absence of racially or ethnically minoritised participants and participants with physical disabilities.*

**Response:** We strengthened the Limitations section to address each of these boundaries and revised the Abstract, Results and Discussion, and Conclusions to use sample-specific and exploratory language. The revised text no longer claims general prevalence or causal operation beyond the institutional setting studied.

**4. Restricted data and governance**

**Comment:** *Specify what materials may be shared, under what conditions, and through which governance procedures.*

**Response:** The revised Data Availability Statement specifies that full transcripts will not be released because of identifiability, consent, and ethics constraints. De-identified excerpts supporting the findings are included in the article and the coding tree is supplied as S3 Appendix. Requests for additional anonymised excerpts may be considered for qualified researchers after Ethics Committee review, confirmation of compatibility with consent and approval, and any required data-use agreement.

**5. Readability and conceptual density**

**Comment:** *Simplify theoretically dense passages in the Introduction and Discussion, reduce conceptual layering, and shorten long sentences.*

**Response:** We simplified the social identity threat paragraph in the Introduction and removed the early SAFE-model layering. The SAFE model is now introduced only in the Conclusions, where its explanation was shortened and divided into two paragraphs. We also divided the construct-differentiation and limitations passages, rewrote the policy implications as parallel complete sentences, and shortened several abstract and discussion sentences.

**Closing**

We appreciate the editor’s and reviewers’ suggestions. We believe the revisions have improved the manuscript’s transparency, conceptual precision, methodological auditability, and accessibility while keeping the claims proportionate to the evidence.

Sincerely,
The authors
