# Supplementary material for: Perceptions of homogeneity reproduction in health sciences academia
Source: PLoS One. 2026 Oct 28;21(10):e0345194. doi: 10.1371/journal.pone.0345194 (PMC13716225; doi:10.1371/journal.pone.0345194)
Supplement: Peer review history

# Peer review: Decision Letter 0

**DOI:** 10.1371/journal.pone.0345194.r001

**Authors:** Jianhong Zhou

22 Jul 2026

Dear Dr. Smith,

Thank you for submitting your manuscript to PLOS ONE. After careful consideration, we feel that it has merit but does not fully meet PLOS ONE’s publication criteria as it currently stands. Therefore, we invite you to submit a revised version of the manuscript that addresses the points raised during the review process.

Please submit your revised manuscript by Sep 05 2026 11:59PM. If you will need more time than this to complete your revisions, please reply to this message or contact the journal office at plosone@plos.org. When you're ready to submit your revision, log on to https://www.editorialmanager.com/pone/ and select the 'Submissions Needing Revision' folder to locate your manuscript file.

- A letter that responds to each point raised by the academic editor and reviewer(s). You should upload this letter as a separate file labeled 'Response to Reviewers'.
- A marked-up copy of your manuscript that highlights changes made to the original version. You should upload this as a separate file labeled 'Revised Manuscript with Track Changes'.
- An unmarked version of your revised paper without tracked changes. You should upload this as a separate file labeled 'Manuscript'.

If you would like to make changes to your financial disclosure, please include your updated statement in your cover letter. Guidelines for resubmitting your figure files are available below the reviewer comments at the end of this letter.

If applicable, we recommend that you deposit your laboratory protocols in protocols.io to enhance the reproducibility of your results. Protocols.io assigns your protocol its own identifier (DOI) so that it can be cited independently in the future. For instructions see: https://journals.plos.org/plosone/s/submission-guidelines#loc-laboratory-protocols. Additionally, PLOS ONE offers an option for publishing peer-reviewed Lab Protocol articles, which describe protocols hosted on protocols.io. Read more information on sharing protocols at https://plos.org/protocols?utm_medium=editorial-email&utm_source=authorletters&utm_campaign=protocols.

As the corresponding author, your ORCID iD is verified in the submission system and will appear in the published article. PLOS supports the use of ORCID, and we encourage all coauthors to register for an ORCID iD and use it as well. Please encourage your coauthors to verify their ORCID iD within the submission system before final acceptance, as unverified ORCID iDs will not appear in the published article. *Only*  the individual author can complete the verification step; PLOS staff *cannot*  verify ORCID iDs on behalf of authors.

We look forward to receiving your revised manuscript.

Kind regards,

Jianhong Zhou

Staff Editor

PLOS One

Journal Requirements:

When submitting your revision, we need you to address these additional requirements.

1.Please ensure that your manuscript meets PLOS ONE's style requirements, including those for file naming. The PLOS ONE style templates can be found at

https://journals.plos.org/plosone/s/file?id=wjVg/PLOSOne_formatting_sample_main_body.pdf and

https://journals.plos.org/plosone/s/file?id=ba62/PLOSOne_formatting_sample_title_authors_affiliations.pdf

2. We note that the grant information you provided in the ‘Funding Information’ and ‘Financial Disclosure’ sections do not match.

When you resubmit, please ensure that you provide the correct grant numbers for the awards you received for your study in the ‘Funding Information’ section.

3. Thank you for stating the following in your Competing Interests section:

“None known”

Please complete your Competing Interests on the online submission form to state any Competing Interests. If you have no competing interests, please state "The authors have declared that no competing interests exist.", as detailed online in our guide for authors at http://journals.plos.org/plosone/s/submit-now

This information should be included in your cover letter; we will change the online submission form on your behalf.

4. Please note that funding information should not appear in any section or other areas of your manuscript. We will only publish funding information present in the Funding Statement section of the online submission form. Please remove any funding-related text from the manuscript.

5. In the online submission form, you indicated that your data is available only on request from a third party. Please note that your Data Availability Statement is currently missing the name of the third party contact or institution / contact details for the third party, such as an email address or a link to where data requests can be made. Please update your statement with the missing information.

6. Your ethics statement should only appear in the Methods section of your manuscript. If your ethics statement is written in any section besides the Methods, please move it to the Methods section and delete it from any other section. Please ensure that your ethics statement is included in your manuscript, as the ethics statement entered into the online submission form will not be published alongside your manuscript.

7. If the reviewer comments include a recommendation to cite specific previously published works, please review and evaluate these publications to determine whether they are relevant and should be cited. There is no requirement to cite these works unless the editor has indicated otherwise.

[Note: HTML markup is below. Please do not edit.]

Reviewer's Responses to Questions

**Comments to the Author**

1. Is the manuscript technically sound, and do the data support the conclusions?

Reviewer #1: Partly

Reviewer #2: Yes

**********

2. Has the statistical analysis been performed appropriately and rigorously?

Reviewer #1: N/A

Reviewer #2: N/A

**********

3. Have the authors made all data underlying the findings in their manuscript fully available?

The PLOS Data policy

Reviewer #1: No

Reviewer #2: Yes

**********

4. Is the manuscript presented in an intelligible fashion and written in standard English?

Reviewer #1: Yes

Reviewer #2: Yes

**********

Reviewer #1: This study addresses an important topic. However, some issues should be addressed to improve the transparency of the study:

1. The reference numbering appears somewhat inconsistent throughout the manuscript (for example, citation [50] appears after [7] in the Introduction). It would be helpful to check that references are numbered sequentially according to their first appearance, in line with journal guidelines.

2. The limitation of snowball sampling is already acknowledged (line 511), but it would be helpful to briefly explain how this might have influenced the findings. For example, it’s possible that the results mainly reflect individuals who are more connected within academic networks.

3. In line 526, the statement “our findings identify exclusion…as the mechanism through which social homogeneity is reproduced in academic environments” feels broader than what the data can support, especially given that the sample does not include racially/ethnically minoritized participants. It would help to revise this sentence, so it better reflects the specific context of the study and avoids overgeneralization.

4. In line 529, the statement that the findings are “consistent with a self-reinforcing cycle” still feels a bit strong given the small and context-specific sample. It might help to present this more cautiously, for example as a possible or exploratory interpretation.

5. The results are well presented. It would further strengthen the manuscript to include a brief example of how the analysis was conducted in practice (for instance, how a specific quote was coded and developed into a theme) in the Data Analysis section. This would help make the analytic process clearer to the reader.

Overall, the manuscript is well developed, and I recommend minor revisions to improve clarity and transparency.

Reviewer #2: The manuscript addresses an important and timely issue in health sciences academia: the reproduction of social homogeneity through institutional norms, interpersonal dynamics, and identity management processes. The topic is highly relevant to ongoing discussions regarding diversity, equity, inclusion, academic culture, and workforce wellbeing. The manuscript is theoretically ambitious and demonstrates strong engagement with sociological, organizational, and social psychological literature.

Overall, the study is methodologically credible, and the conclusions are generally supported by the qualitative data presented. The use of reflexive thematic analysis within an interpretivist framework is appropriate for the research aims, and the manuscript demonstrates transparency regarding recruitment, reflexivity, coding, and analytic procedures. The integration of participant quotations throughout the findings section effectively supports the identified themes.

One of the manuscript’s strongest contributions is the conceptualization of “anticipatory compliance” as a mechanism linking conformity pressure, identity threat, and self-exclusion. This concept has potential value for understanding how individuals adapt to exclusionary institutional cultures. However, the construct currently overlaps conceptually with several existing frameworks, including covering, passing, self-surveillance, impression management, and minority stress processes. Although the authors acknowledge some of these overlaps, the manuscript would benefit from a more explicit clarification of what is analytically novel about anticipatory compliance and how it differs from related constructs. Strengthening construct differentiation would improve conceptual precision and enhance the manuscript’s theoretical contribution.

A second concern relates to the interpretive reach of the findings. Although the manuscript appropriately frames itself as qualitative and exploratory, some sections move quickly from participant perceptions to broader structural conclusions. The integration of external statistical evidence within the findings section occasionally creates ambiguity regarding what derives directly from participant narratives versus broader empirical literature. Stronger separation between participant perception, theoretical interpretation, and structural inference would improve analytic clarity and reduce the risk of overstating conclusions from a small qualitative sample.

The study’s limitations are acknowledged appropriately, particularly regarding the small sample size, snowball sampling strategy, and limited representation across diversity domains. The absence of racially or ethnically minoritized participants and participants with physical disabilities substantially limits the manuscript’s ability to speak comprehensively about multiple forms of exclusion. In addition, the faculty-heavy sample composition may privilege institutional or “enacting” perspectives over experiences of early-career vulnerability. While the authors acknowledge these issues transparently, the manuscript should maintain caution in extending claims beyond the specific institutional context studied.

The qualitative methodology itself is generally rigorous. The reflexivity section is particularly strong and demonstrates thoughtful engagement with positionality and interpretive responsibility. The explanation of reflexive thematic analysis is consistent with contemporary qualitative methodological standards. Importantly, the authors avoid inappropriate quantification of qualitative findings and do not make overtly causal claims unsupported by the study design.

Regarding data availability, the ethical rationale for restricting public access to interview transcripts is understandable given the small sample size, single-institution setting, and sensitive nature of the topic. However, the current Data Availability Statement would benefit from greater specificity regarding what materials may be shared, under what conditions, and through which governance procedures. PLOS ONE generally expects clearer procedural details for restricted-access qualitative datasets. The authors should clarify whether partially anonymized excerpts, coding frameworks, or analytic materials could be made available through controlled-access procedures or institutional repositories.

The manuscript is generally well written and presented in strong academic English. The writing is intellectually sophisticated and coherent. However, several sections particularly within the Introduction and Discussion, are theoretically dense and occasionally difficult to follow. Some paragraphs introduce multiple conceptual frameworks simultaneously, reducing readability for interdisciplinary audiences. Simplifying overly abstract language, reducing conceptual layering within individual paragraphs, and shortening several long sentences would improve accessibility without reducing intellectual depth.

I did not identify major concerns regarding research misconduct, dual publication, or ethical violations. Ethical approval, informed consent procedures, and reflexivity considerations are adequately described.

Overall, this manuscript has clear scholarly merit and strong publication potential. The topic is important, the theoretical framing is thoughtful, and the qualitative findings contribute meaningfully to discussions of exclusion and belonging within academia. However, the manuscript would benefit from revisions that improve conceptual precision, strengthen boundaries between interpretation and structural inference, clarify the novelty of key constructs, and enhance readability for a broader public health audience.

Recommendation: Major Revision.

**********

what does this mean?). If published, this will include your full peer review and any attached files.

If you choose “no”, your identity will remain anonymous but your review may still be made public.

**Do you want your identity to be public for this peer review?**  For information about this choice, including consent withdrawal, please see our Privacy Policy

Reviewer #1: No

Reviewer #2: **Yes:**  Rupam Mitra

**********

[NOTE: If reviewer comments were submitted as an attachment file, they will be attached to this email and accessible via the submission site. Please log into your account, locate the manuscript record, and check for the action link "View Attachments". If this link does not appear, there are no attachment files.]

To ensure your figures meet our technical requirements, please review our figure guidelines: https://journals.plos.org/plosone/s/figures

You may also use PLOS’s free figure tool, NAAS, to help you prepare publication quality figures: https://journals.plos.org/plosone/s/figures#loc-tools-for-figure-preparation.

NAAS will assess whether your figures meet our technical requirements by comparing each figure against our figure specifications.

# Author comment: Author response to Decision Letter 1

**DOI:** 10.1371/journal.pone.0345194.r002

6 Aug 2026

Thank you so much for engaging with our paper. We have attached a document with repsonses to each piece of feedback.

Attachment Submitted filename: PONE-D-26-10324_R1_Response_to_Reviewers.docx

# Peer review: Decision Letter 1

**DOI:** 10.1371/journal.pone.0345194.r003

**Authors:** Eyal Bar-Haim

2 Sep 2026

Perceptions of homogeneity reproduction in health sciences academia

PONE-D-26-10324R1

Dear Dr. Smith,

We’re pleased to inform you that your manuscript has been judged scientifically suitable for publication and will be formally accepted for publication once it meets all outstanding technical requirements.

Within one week, you’ll receive an e-mail detailing the required amendments. When these have been addressed, you’ll receive a formal acceptance letter and your manuscript will be scheduled for publication.

An invoice will be generated when your article is formally accepted. Please note, if your institution has a publishing partnership with PLOS and your article meets the relevant criteria, all or part of your publication costs will be covered. Please make sure your user information is up-to-date by logging into Editorial Manager at Editorial Manager® and clicking the ‘Update My Information' link at the top of the page. For questions related to billing, please contact billing support.

If your institution or institutions have a press office, please notify them about your upcoming paper to help maximize its impact. If they’ll be preparing press materials, please inform our press team as soon as possible -- no later than 48 hours after receiving the formal acceptance. Your manuscript will remain under strict press embargo until 2 pm Eastern Time on the date of publication. For more information, please contact onepress@plos.org.

Kind regards,

Eyal Bar-Haim

Academic Editor

PLOS One

Additional Editor Comments (optional):

Reviewers' comments:

Reviewer's Responses to Questions

**Comments to the Author**

Reviewer #1: All comments have been addressed

Reviewer #2: All comments have been addressed

**********

2. Is the manuscript technically sound, and do the data support the conclusions?

Reviewer #1: Yes

Reviewer #2: Yes

**********

3. Has the statistical analysis been performed appropriately and rigorously?

Reviewer #1: N/A

Reviewer #2: Yes

**********

4. Have the authors made all data underlying the findings in their manuscript fully available?

The PLOS Data policy

Reviewer #1: No

Reviewer #2: Yes

**********

5. Is the manuscript presented in an intelligible fashion and written in standard English?

Reviewer #1: Yes

Reviewer #2: Yes

**********

Reviewer #1: This revision responds thoroughly to all prior editor and reviewer comments.

The reflexive thematic analysis and idiographic case engagement are appropriate for the research questions, and sample adequacy is justified via Malterud et al.'s information-power framework. This revision adds a clear coding audit-trail example, improving analytic transparency.

Most importantly, the findings are now appropriately hedged: the proposed "self-reinforcing cycle" is explicitly framed as an exploratory interpretation, not a causal claim, and external statistical evidence is consistently labeled as contextualization rather than corroboration. This directly addresses prior concerns about overgeneralization from a nine-participant, single-institution sample.

Limitations (snowball sampling bias, absence of racially/ethnically minoritized and disabled participants, faculty-heavy sample, 55% non-response) are candidly addressed. The construct of "anticipatory compliance" is now clearly differentiated from related concepts (covering, passing, minority stress, self-surveillance).

Reviewer #2: The articled was modified according to the comments provided by the reviewers and now ready for publications.

**********

what does this mean?). If published, this will include your full peer review and any attached files.

If you choose “no”, your identity will remain anonymous but your review may still be made public.

**Do you want your identity to be public for this peer review?**  For information about this choice, including consent withdrawal, please see our Privacy Policy

Reviewer #1: **Yes:**  Mahsa Pashaeimeykola

Reviewer #2: **Yes:**  Rupam Mitra

**********

# Editor report: Acceptance letter

**DOI:** 10.1371/journal.pone.0345194.r004

**Authors:** Eyal Bar-Haim

PONE-D-26-10324R1

PLOS One

Dear Dr. Smith,

I'm pleased to inform you that your manuscript has been deemed suitable for publication in PLOS One. Congratulations! Your manuscript is now being handed over to our production team.

At this stage, our production department will prepare your paper for publication. This includes ensuring the following:

* All references, tables, and figures are properly cited

* All relevant supporting information is included in the manuscript submission,

* There are no issues that prevent the paper from being properly typeset

You will receive further instructions from the production team, including instructions on how to review your proof when it is ready. Please keep in mind that we are working through a large volume of accepted articles, so please give us a few days to review your paper and let you know the next and final steps.

Lastly, if your institution or institutions have a press office, please let them know about your upcoming paper now to help maximize its impact. If they'll be preparing press materials, please inform our press team within the next 48 hours. Your manuscript will remain under strict press embargo until 2 pm Eastern Time on the date of publication. For more information, please contact onepress@plos.org.

You will receive an invoice from PLOS for your publication fee after your manuscript has reached the completed accept phase. If you receive an email requesting payment before acceptance or for any other service, this may be a phishing scheme. Learn how to identify phishing emails and protect your accounts at https://explore.plos.org/phishing.

If we can help with anything else, please email us at customercare@plos.org.

Thank you for submitting your work to PLOS One and supporting open access.

Kind regards,

PLOS One Editorial Office Staff

on behalf of

Dr. Eyal Bar-Haim

Academic Editor

PLOS One
